# USDA-ARS Colorado maize growth and development, yield and water-use under strategic timing of irrigation, 2012–2013

**DOI:** 10.1016/j.dib.2018.10.140

**Published:** 2018-10-30

**Authors:** Louise H. Comas, Thomas J. Trout, Garrett T. Banks, Huihui Zhang, Kendall C. DeJonge, Sean M. Gleason

**Affiliations:** USDA-ARS Water Management and Systems Research, Ft. Collins, CO, USA

## Abstract

This data set was collected over two years, 2012–2013, on maize under 12 irrigation treatments with varying levels of deficit during late-vegetative and grain-filling growth stages in semi-arid Northern Colorado supplied with surface drip irrigation. The data set, which can be found online at the USDA National Agricultural Library data repository (doi: 10.15482/USDA.ADC/1439968), includes hourly weather data; plant growth and canopy development over the season; final biomass, yield and harvest index; and daily water balance data including irrigation, precipitation, soil water content, and estimates of crop evapotranspiration. Soil parameters for the site, as well as data from a previous experiment on maize with different treatments can also be found online (doi: 10.15482/USDA.ADC/1254006). Here, we describe the synthesis of data collected from 2012 to 2013. These data can be used for modeling the relationship between maize yield and field-level water use under season water availability.

**Specifications table**

**Table:** 

Subject area	*Biology, Agricultural engineering*
More specific subject area	*Crop ecophysiology, Agronomy, Irrigation management*
Type of data	*Table, figure*
How data was acquired	*Observation, destructive sampling, weather station (*https://coagmet.colostate.edu/station_description.php*; GLY04), neutron soil moisture meter (CPN-503DR Hydroprobe, InstroTek, San Francisco, CA USA), portable time domain reflectometer (TDR, Minitrase, Soil-moisture Equipment Corp, Santa Barbara, CA, USA), leaf area meter (LI-3100C; LI-COR, Lincoln, Nebraska, USA), RGB camera (EOS 50D; Canon, Oita, Japan).*
Data format	*Raw and calculated*
Experimental factors	*Irrigation deficits were applied independently during two stress periods corresponding with late vegetative (V8-VT) and grain-filling (R4-R6) stages. Stress periods were chosen to avoid stress early in plant development (prior to V7) and during anthesis (R1 until R4).*
Experimental features	*Data were collected from field plots of maize that were 9 m wide (12 rows) by 43 m long. Twelve irrigation treatments were laid out in a complete block design with four blocks.*
Data source location	*N.E. of Greeley Colorado; 40°26’ N, 104°38’ W*
Data accessibility	*Available for download at the USDA National Agricultural Library data repository (*DOI: 10.15482/USDA.ADC/1439968*;*https://data.nal.usda.gov/dataset/usda-ars-colorado-maize-water-productivity-dataset-2012*–2013)*
Related research article	*Comas LH, Trout TJ, DeJonge KC, Zhang H, Gleason SM. In press. Water productivity under strategic growth stage-based deficit irrigation in maize. Agricultural Water Management*

**Value of the data**

• The data provide maize growth, development, yield and evapotranspiration (ET, i.e. consumptive use of water from the crop system) with restricted water availability at different times of the season (late-vegetative and grain-filling periods).
• The data can be used to parameterize and/or validate models on crop production and water use.
• The data can be used to calculate crop water use efficiency and examine environmental and managerial effects on crop productivity.

## 1 Data

**Fig. 1:**
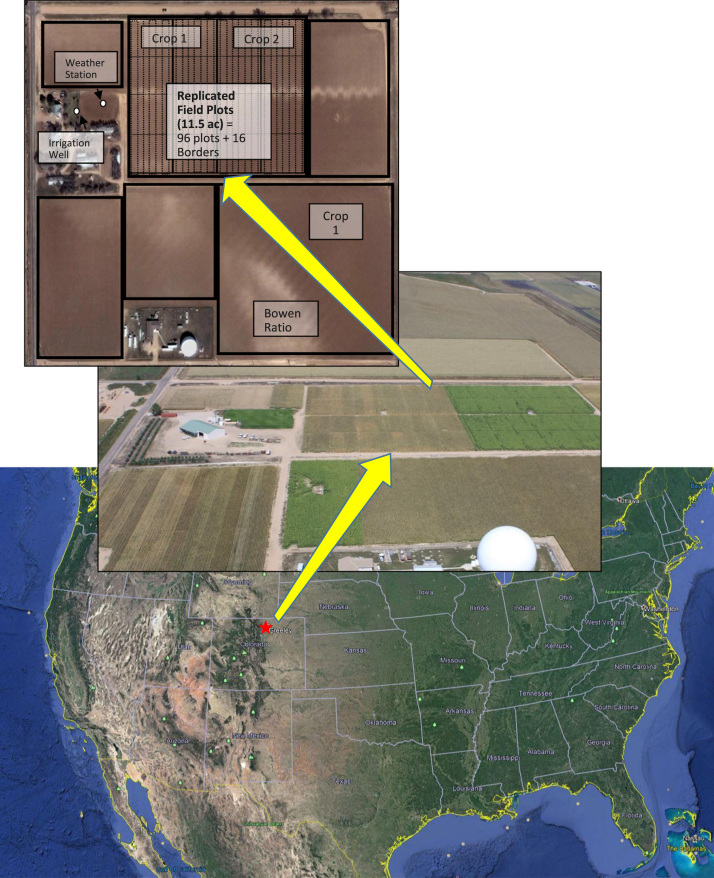
Map of the Limited Irrigation Research Farm and plots. The red star indicates the location of the research farm (LIRF).

Data on maize growth, canopy development, yield and ET were gathered from field plots in a semi-arid crop research farm located in Weld County, which is the most productive agricultural county in Colorado and the ninth most productive in the US (Fig. 1) [1]. Data were collected from large plots, approximately 43 m long by 9 m wide. Raw data were included, where useful independently, in addition to the data derived from this raw data. Raw meteorological data are available elsewhere (http://www.coagmet.com, station GLY04) but included here after being subjected to additional quality protocols and filling in missing data from nearby weather stations.

## 2 Experimental design, materials, and methods

Data on maize growth, development, yield and ET were gathered from field plots located at the USDA-ARS Limited Irrigation Research Farm (LIRF) located near Greeley, CO USA (40°26′50″N, 104°38′12″W, 1425 m elevation), which receives approximately 215 mm of precipitation during the growing season (May–October). These data were collected from 12 irrigation treatments replicated in individual plots within each of four blocks and rotated between two fields among years (a total of 48 plots each year). Irrigations were applied every 4–5 d to meet a percentage of crop ET requirements during target growth stages.

**Figure S1:**
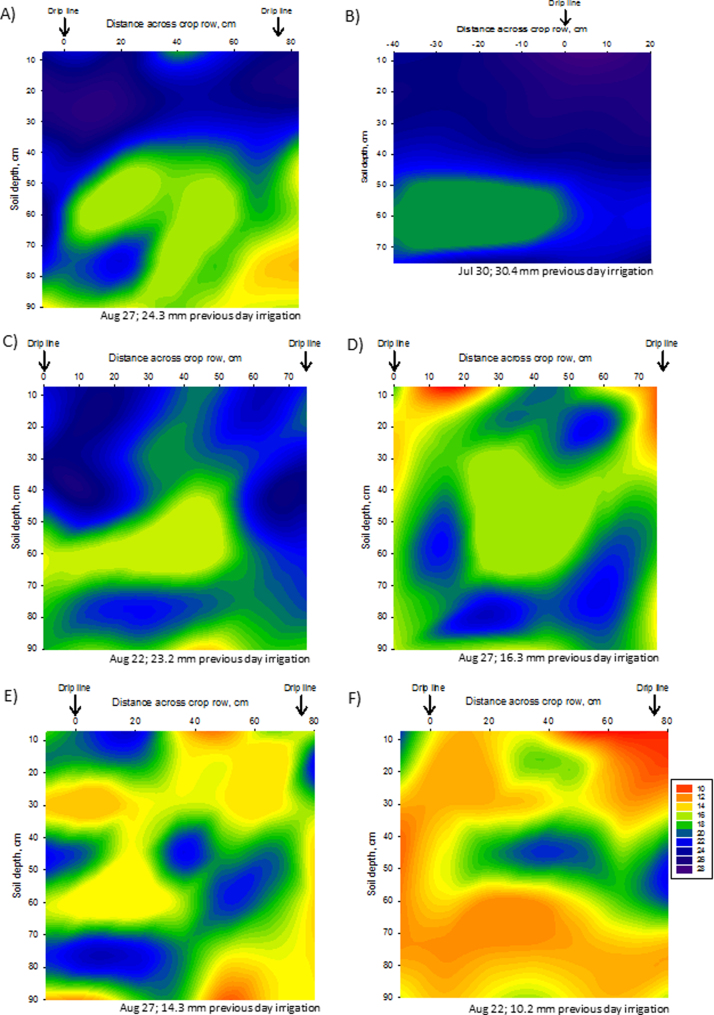
Maps of soil moisture content (percent by volume) measured with a portable time domain reflectometer (TDR, Minitrase, Soil-moisture Equipment Corp, Santa Barbara, CA, USA) in soil pits excavated in 2013 in 100% (A, B), 65% (C, D) and 40% (E, F) ET treatments between two rows down to 1 m. TDR was measured on the face of the wall in 15 cm increments across and down with contour lines created in SigmaPlot 13.0 (Systat Software, Inc., San Jose, CA, USA). Two soil pits, each in two different plots, were measured for the 100% ET target. Measurements after two different irrigations from the same pits were measured for the 65 and 40% ET targets.

Data collection is described in detail elsewhere [2]. Agronomical information was documented. Canopy cover was determined from nadir digital images collected twice weekly through the irrigation season until full cover was reached. Leaf area index (LAI) was collected destructively four times through the season. Plant height was collected twice a week in 2012 and every 1–2 weeks in 2013 until the plants tasseled. Meteorological data are provided hourly. Soil moisture was collected before and after each irrigation and used in combination with crop and meteorological data to calculate and verify crop ET through water balance. All data were screened and a few removed when plots had poor stand or when equipment was not working correctly. Soil pits were dug perpendicular to two rows in 2013. Two-dimensional soil moisture profiles were mapped to determine if soil moisture measurements collected near the drip lines were representative of the horizontal distribution as needed for calculating an accurate water balance (Fig S1). Additional economic parameters calculated using these data are given elsewhere [3]. The experiment that supported this data collection also supported other data collection, such as infrared thermometry and sap flow, and crop modeling presented elsewhere [4], [5], [6], [7], [8], [9], [10], [11], [12], [13].
