# Supplementary material for: USDA-ARS Colorado maize growth and development, yield and water-use under strategic timing of irrigation, 2012–2013
Source: Data Brief. 2018 Oct 30;21:1227–31. doi: 10.1016/j.dib.2018.10.140 (PMC6231082; doi:10.1016/j.dib.2018.10.140)
Supplement: Supplementary file 1 — Supplementary material [file mmc1.docx]

**Conflict of interest**

Disclosure is accomplished in three ways:

First, by a complete listing of the current institutional affiliations of the authors. This list must include academic as well as corporate and other industrial affiliations. As the editors deem appropriate, items in this list will be included in the author affiliations printed in the manuscript. Please indicate below:

____All my affiliations are listed on the title page of the paper.

__x__Additional affiliations not on the title page are listed below:

Affiliated faculty, Colorado State University

Second, through the acknowledgment of all financial contributions to the work being reported, including contributions "in kind." All funding sources will be listed in the published manuscript. Please indicate below:

__x__All my funding sources for this study are listed in the acknowledgement section of the paper.

____Additional funding sources not noted in the manuscript are listed below:

Third, through the execution of a statement disclosing to the Editors all financial holdings, professional affiliations, advisory positions, board memberships, patent holdings and the like that might bear a relationship to the subject matter of the contribution. The Editors will determine whether the material disclosed to them should be published as part of the article.

The following are declarable relationships:

Financial: Significant financial interest (equity holdings or stock options) in any corporate entity dealing with the material or the subject matter of this contribution. Please disclose the entity and the nature and amount of the holding:

__x___None

____I have a financial relationship, as described below.

Management/Advisory affiliations: Within the last 3 years, status as an officer, a member of the Board, or a member of an Advisory Committee of any entity engaged in activity related to the subject matter of this contribution. Please disclose the nature of these relationships and the financial arrangements.

_____None

____I have a management/advisory relationship, as described below:

Paid Consulting: Within the last 3 years, receipt of consulting fees, honoraria, speaking fees, or expert testimony fees from entities that have a financial interest in the results and materials of this study. Please enumerate.

_____None

____I have a consulting relationship, as described below:

Patents: A planned, pending, or awarded patent on this work by any of the authors or their institutions. Please explain.

_____None

____I or my institution has a patent related to this work, as described below

Declaration: By completing this form, I declare that I have disclosed all declarable relationships as defined therein, if any.

This form was completed on ___Oct 19, 2018___________________

Signature_________________________________

Name___Louise Comas_________________________________
